# A Clinical Text-Book of Surgical Diagnosis and Treatment

**Published:** 1898-12

**Authors:** 


**A Clinical Text-Book of Surgical Diagnosis and Treatment.** By J. W. MACDONALD, M.D. Pp. 798. London: The Rebman Publishing Co. (Ltd.). Philadelphia: W. B. Saunders. 1898.

This book is devoted entirely to diagnosis and treatment, without touching at all upon the principles of surgery, surgical pathology, or bacteriology. We question if it will prove of much service to students; but to the young practitioner it ought to be of great value, inasmuch as it will enable him to possess in a single volume of moderate size the most practical part of practical surgery. Dr. Macdonald, who is a graduate of Edinburgh University, is professor of the practice of surgery in Hamline University, Minneapolis, and he is able to give his readers all that is best known of the practice of surgery on both sides of the Atlantic. We notice very few signs of original work. The illustrations are mostly taken from well-known text-books, and the methods of treatment advised by the author are those favoured by most modern surgeons. In a work such as this, designed to give in a small compass all that is best known and approved, this want of originality is scarcely a fault, and inasmuch as good sense and caution have been exercised in choosing all that is good and rejecting all that is mischievous in the work of others, we are presented with a book which may be safely followed, and which may be used as a work of reference by the young surgeon.

